# Oxyplasma meridianum gen. nov., sp. nov., an extremely acidophilic organotrophic member of the order Thermoplasmatales

**DOI:** 10.1099/ijsem.0.006499

**Published:** 2024-08-27

**Authors:** Olga V. Golyshina, Evgenii A. Lunev, Marco A. Distaso, Rafael Bargiela, Matthew C. Gaines, Bertram Daum, Manuel Ferrer, Nicole J. Bale, Michel Koenen, Jaap S. Sinninghe Damsté, Mikhail M. Yakimov, Peter N. Golyshin

**Affiliations:** 1 Centre for Environmental Biotechnology, School of Environmental and Natural Sciences, Bangor University, Bangor, UK; 2 Instituto de Catalisis y Petroleoquimica (ICP), CSIC, Madrid, Spain; 3 Living Systems Institute and Department of Biosciences, Faculty of Health and Life Sciences, University of Exeter, Exeter, UK; 4 Department of Marine Microbiology and Biogeochemistry, NIOZ Royal Netherlands Institute for Sea Research, Texel, Netherlands; 5 Institute of Polar Sciences, CNR, Messina, Italy

**Keywords:** acidic environments, acidophilic archaea, geothermal environments, *Oxyplasma*, *Thermoplasmatales*

## Abstract

A mesophilic, hyperacidophilic archaeon, strain M1^T^, was isolated from a rock sample from Vulcano Island, Italy. Cells of this organism were cocci with an average diameter of 1 µm. Some cells possessed filaments. The strain grew in the range of temperatures between 15 and 52 °C and pH 0.5–4.0 with growth optima at 40 °C and pH 1.0. Strain M1^T^ was aerobic and chemoorganotrophic, growing on complex substrates, such as casamino acids, trypticase, tryptone, yeast and beef extracts. No growth at expenses of oxidation of elemental sulphur or reduced sulphur compounds, pyrite, or ferrous sulphate was observed. The core lipids were glycerol dibiphytanyl glycerol tetraether lipids (membrane spanning) with 0 to 4 cyclopentane moieties and archaeol, with trace amounts of hydroxy archaeol. The dominant quinone was MK-7 : 7. The genome size of M1^T^ was 1.67 Mbp with a G+C content of 39.76 mol%, and both characteristics were well within the common range for *Thermoplasmatales*. The phylogenetic analysis based on 16S rRNA gene sequence placed the strain M1^T^ within the order *Thermoplasmatales* with sequence identities of 90.9, 90.3 and 90.5% to the closest SSU rRNA gene sequences from organisms with validly published names, *Thermoplasma acidophilum*, *Thermoplasma volcanium* and *Thermogymnomonas acidicola*, respectively. Based on the results of our genomic, phylogenetic, physiological and chemotaxonomic studies, we propose that strain M1^T^ (=DSM 116605^T^=JCM 36570^T^) represents a new genus and species, *Oxyplasma meridianum* gen. nov., sp. nov., within the order *Thermoplasmatales*.

## Introduction

Archaea of the order *Thermoplasmatales* are ubiquitous across most terrestrial acidic environments of various scale and origin (geothermal and anthropogenic sulphide-ore-containing mining biotopes with temperatures between 10 and 60 °C) [1,3]. These archaea occur in a variety of sites in significant abundance, suggesting they contribute substantially to element cycling and community composition [4,6]. However, most of these archaea were detected by obtaining metagenomes worldwide and, thus, remain uncultured. Up to now, there are six genera with validly published names within the order *Thermoplasmatales*: *Thermoplasma, Picrophilus, Ferroplasma*, *Thermogymnomonas*, *Acidiplasma* and *Cuniculiplasma* [7]. The scarcity of isolated and described members limits our knowledge about the ecological, physiological, morphological, and chemotaxonomic properties of these organisms. The phylogenetic position of the order *Thermoplasmatales* has been recently changed. Originally, the order was affiliated with the phylum *Euryarchaeota* [2]. However, an updated phylogenetic reconstruction from Genome Taxonomy Database (GTDB) firmly placed these organisms as a separate phylum, namely *Thermoplasmatota* [8,9]. According to this classification, the phylum *Thermoplasmatota* comprises classes ‘*Ca*. Poseidonia’ and *Thermoplasmata*, with the latter containing multiple orders along with *Thermoplasmatales*. The tight clustering of *Thermoplasmatales* together with other orders into a separate phylum point at a distinct evolutionary trajectory for this group of organisms. There are also other factors, making these archaea particularly attractive for further isolation efforts and study. Archaea of the order *Thermoplasmatales* are the most acidophilic organisms among prokaryotes, able to survive at pH values lower than 0 [10]. Another hallmark is the lack of cell walls in most cultured members, leading to a pleomorphic morphology, which is unusual for archaea [2]. Finally, these hyperacidophilic archaea are an attractive target for bioprospecting of enzymes and metabolites of biotechnological relevance [11,15].

In this study, we describe a new member of the order *Thermoplasmatales* isolated from a rock sample of Vulcano Island, Italy. Based on the morphological, physiological, chemotaxonomic, and phylogenetic characteristics, the organism represents a new genus and species within the order *Thermoplasmatales*.

## Methods

### Sampling, isolation and cultivation conditions

The sampling was conducted in the Levante Bay of Vulcano Island (Aeolian archipelago, Italy; 38.416115° N 14.96035° E) in October 2012. The collected sample was a soft biofilm on the surface of the rock. For cultivation, the modified medium DSMZ 88 was used, which contained (g l^−1^): (NH_4_)_2_SO_4_, 1.3; KH_2_PO_4_, 0.28; MgSO_4_·7H_2_O, 0.25; CaCl_2_·2H_2_O, 0.07; FeCl_3_·6H_2_O, 0.02. Additionally, the medium was amended with the trace element solution SL-10 from DSMZ medium 320 in proportion 1 : 1000 (v/v), betaine at 0.06% (w/v) and vitamin solution (Kao and Michayluk, Sigma-Aldrich) at 1 : 100 (v/v). Beef extract and tryptone (both ThermoFisher Scientific), were added at a final concentration of 1 g l^−1^. Casamino acids, trypticase, yeast extract, amino acids, casein, chitin, cellulose, sucrose, lactose, raffinose, xylose, glucose and galactose (all from ThermoFisher Scientific) were individually tested at a concentration of 1 g l^−1^. The pH of the medium was adjusted to pH 1.0–1.2 with concentrated H_2_SO_4_. The oxidation of FeSO_4_·7H_2_O (25 g l^−1^) and pyrite (Kremer Pigments; 1 g l^−1^) were tested in the medium DSMZ 874, as described previously, in the presence of 0.02% (w/v) yeast extract (ThermoFisher Scientific) [16]. Reduced sulphur compounds, K_2_S_4_O_6_ and K_2_S_2_O_3_ (both 5 mM), and 1 g l^−1^ elemental sulphur (Sigma Aldrich) were tested separately in DSMZ medium 88 in the presence of beef extract and tryptone (1 g l^−1^). Anaerobic growth with Fe_2_(SO_4_)_3_ (10 mM) with and without yeast extract addition (0.02% w/v) was studied in DSMZ media 88 and 874. Furthermore, the consumption of H_2_ (1 and 5 ml taken by syringe from gas phase) was tested with and without Fe_2_(SO_4_)_3_ (10 mM) under anaerobic conditions in DSMZ medium 88. Growth under anaerobic conditions was studied also in the presence of elemental sulphur (1 g l^−1^), ferric citrate (1 mM) and KNO_3_ (10 mM), and fermentative growth was tested in the DSMZ medium 88 with addition of beef extract and tryptone (both in concentrations 1 g l^−1^). The gas mixture used for anaerobic atmosphere was N_2_:H_2_:CO_2_, in proportion 80 : 10 : 10. Anaerobic growth was studied in 18 ml Hungate type culture tubes with butyl rubber stoppers (Glasgerätebau Ochs) with the volume of cultures between 5 and 15 ml and with the gas headspace varied between 3 and 13 ml. The pure culture was isolated by the serial dilution to extinction method and monitored by PCR amplification with universal bacterial (F27 and R1492) and archaeal (AF23 and R1492) primers. The purified cultures were grown aerobically in 100 ml Erlenmeyer borosilicate Duran flasks (DWK Life Sciences) for ca. 5 days with 25 ml medium and shaking at 120 r.p.m. Growth was studied at between 5 and 55 °C and pH between 0 and 4.5. The modified DSMZ medium 88 with amendments described above and organic substrates (beef extract and tryptone, both in concentrations 1 g l^−1^) with various pH values was used for determining the optimal pH for growth. The growth was estimated spectrophotometrically at the wavelength 600 nm in a BioPhotometer Plus (Eppendorf), and direct cell counts in a Thoma chamber for anaerobic cultivation conditions.

### Cell morphology

Cell morphology was investigated with the use of TEM. For TEM, 5 µl liquid cell culture was pipetted onto Cu 400 mesh negative stain grids (Agar Scientist AGS160-4). These grids had been glow discharged at 20 mA for 1 min and the biological sample applied within 15 min after glow discharging. The sample was left on the grid for 2 min, before excess liquid was blotted off from the periphery of the grid using filter paper (Whatman, Grade 1). Three wash steps using Milli-Q water were performed, whereby 5 µl Milli-Q was pipetted onto the grid before being immediately removed using filter paper via the same blotting method. 1% uranyl acetate stain (dissolved in Milli-Q) was then applied to the grid, before being immediately blotted off. Finally, a second application of 1% uranyl acetate stain was pipetted onto the grid and left for 30 s before blotting. The grids were then left to dry on filter paper for 20 min before being investigated in a Thermo Fisher Tecnai Spirit TEM, operating at 120 kV. Images were recorded using a OneView CMOS detector (Gatan).

### Chemotaxonomic characterization

The intact polar lipids (IPLs) and quinones were extracted from freeze-dried biomass with methanol, dichloromethane (DCM) and phosphate buffer (2 : 1 : 0.8, v:v:v) using an ultrasonic bath (2×10 min). The extracts were phase-separated by adding DCM and buffer to a final solvent ratio of 1 : 1 : 0.9 (v:v:v). The IPL-containing organic phases were re-extracted twice with DCM. All steps of the extraction were then repeated on the freeze-dried biomass with a solvent mixture of methanol, DCM and trichloroacetic acid pH 2–3 (2 : 1 : 0.8, v:v:v). Finally, the combined extract was dried under a stream of N_2_ gas [17]. For analysis, the extracts were redissolved in MeOH:DCM (9 : 1, v:v) and filtered through 0.45 µm cellulose syringe filters (4 mm diameter; Grace Alltech). Analysis was carried out using ultra high-pressure liquid chromatography–high resolution mass spectrometry [17]. Identification was carried out by comparison of accurate masses and mass spectral fragmentation with published data for IPLs and for quinones [18,20].

### Genome sequencing, annotation and comparative genomic analysis

The DNA was isolated using a Qiagen DNeasy PowerLyzer PowerSoil Kit according to the manufacturer’s protocol using 25–100 ml cultures and quantified using Qubit dsDNA BR Assay kit and Qubit fluorometer (Invitrogen). The genome was sequenced using in-house Illumina MiSeq and Nanopore platforms. Pre-processing of Nanopore reads were conducted by porechop (https://github.com/rrwick/Porechop) and filtlong (https://github.com/rrwick/Filtlong). The assembly was performed using the ‘zga’ pipeline (https://github.com/laxeye/zga) with Unicycler version 0.4.4. Genome annotation was performed using PGAP version 2022-12-13.build6494 [21] and the genome was submitted to GenBank with accession number CP133772. Comparative genomic analysis was done using a dDDH (digital DNA–DNA hybridization) calculation, formula *d4* and average nucleotide identity (ANIb and ANIm), utilizing the DSMZ platform (https://tygs.dsmz.de) and the JSpeciesWS web server (https://jspecies.ribohost.com/jspeciesws/#analyse), respectively [22,23].

### Phylogenetic analysis

For phylogenetic analysis based on 16S rRNA gene sequences, reference sequences were downloaded from the NCBI nucleotide database and aligned using mafft version 7 [24]. Multiple alignment was trimmed using TrimAL 1.2rev59 [25] and the tree was reconstructed by the maximum-likelihood method with a bootstrap based on 1000 replicates using the R package phangorn [26]. Graphical representation for both phylogenetic trees was developed using R programming language [27] and the ape package [28]. Phylogenetic analysis based on 122 proteins alignment was performed using GTDB-tk tool v2.1.1 [29].

## Results and discussion

### Phenotypic properties

Growth of isolate M1^T^ occurred between 15 and 52 °C with the optimum at 40 °C (doubling time 19.2 h), reaching up to 7×10^8^ cells ml^−1^. Growth occurred in a pH range between 0.5 and 4 with pH 1 being the optimal value. The highest growth rate as determined by optical density measurements was detected with a mixture of beef extract and tryptone in a concentration of 1 g l^−1^ each. The following substrates (1 g l^−1^ each) were stimulating growth at optimal temperature and pH: casamino acids, trypticase, yeast extract, with weak growth detected with amino acids. No growth stimulation was observed with casein, chitin, cellulose, sucrose, lactose, raffinose, xylose, glucose and galactose. No growth or stimulation of growth was observed with addition of elemental sulphur or reduced sulphur compounds (tetrathionate or thiosulfate). No growth on ferrous iron sulphate or pyrite was detected in the presence or absence of yeast extract. No fermentative growth, and no anaerobic growth with any acceptors tested was observed.

### Cell morphology

**Fig. 1.**
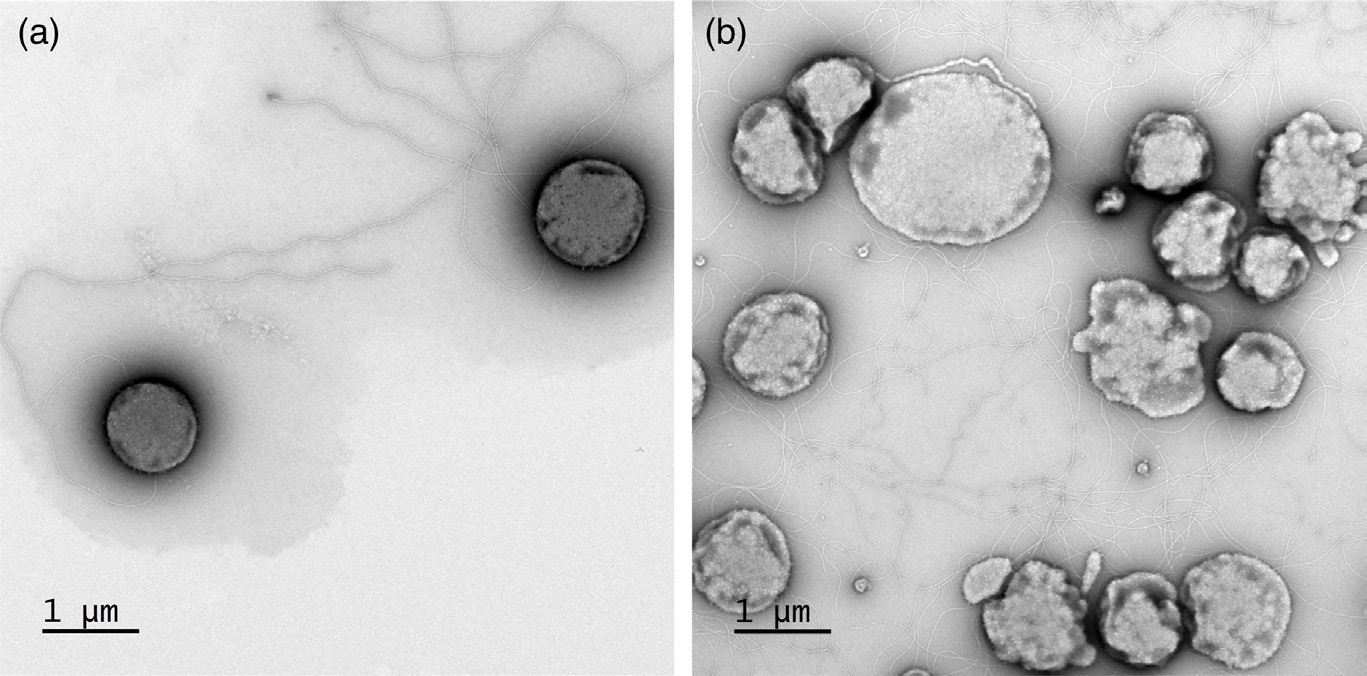
(a, b) Negative stain transmission electron microscopy of strain M1^T^. Microscope images of the cellular periphery of strain M1^T^ cells imaged at different magnifications.

Transmission electron microscopy revealed that cells had slightly irregular coccoid morphology with an average diameter of ~1 µm (Fig. 1). The cells extended on average up to three surface filaments per cell in early log phase. This value then increased to eight surface filaments per cell in the late log to stationary growth phases. These filaments closely resembled pili or archaella. In accordance with previous observations of *Thermoplasmatales* species [2], no canonical S-layer was evident. Notably, the cells were speckled with small (10–20 nm) globular surface structures (Fig. 1). Close inspection of these extensions did not reveal any similarity to viruses and indeed, viral DNA was absent in the culture.

### Chemotaxonomy

For lipid analysis, three separate culture of strain M1^T^ were grown and harvested at the late log phase. The most abundant core lipids (ca. 80%) were glycerol dibiphytanyl glycerol tetraether lipids (GDGTs, membrane spanning) with 0–4 cyclopentane moieties (GDGT-0–GDGT-4). The most dominant GDGTs were GDGT-2 and GDGT-4 (Table 1). A similar GDGT distribution was observed for the (hyper)acidophiles ‘*Ferroplasma acidarmanus*’ [30], *Thermogymnomonas acidicola* [31], *Thermoplasma acidophilum* [32], *Acidiplasma aeolicum* [16], *Cuniculiplasma divulgatum* [7], all also belonging to the order *Thermoplasmatales*. The majority of GDGT IPLs had a phosphoglycerol (PG) head group at one glycerol moiety, with predominantly 1, and to a minor extent, 2–3 hexose sugar(s) (glycosyl; gly) at the other glycerol moiety (Table 2). Archaeol (AR) and minor amounts of hydroxy archaeol (OH-AR) represented the other 20% of the core lipids. PG was the only polar head group of AR detected, whilst OH-AR was only detected with a DiGly head group. The dominant (ca. 94%) quinone present in M1^T^ was MK-7 : 7 (Table 2).

**Table 1.** Intact polar lipids identified in *Oxyplasma meridianum* strain M1^T^ and their relative abundance (in percent of lipid peak area) Columns 1–3 represent the data of the three parallel cultures. Δ mmu, measured mass – calculated mass) × 1000 as calculated for strain 1; AEC, assigned elemental composition; PG, phosphatidylglycerol; Gly, monoglycosyl; diGly, diglycosyl; triGly, triglycosyl; AR, archaeol; OH-AR, hydroxy archaeol; GDGT, glycerol dibiphytanyl glycerol tetraether.

Polarheadgroup 1	Polarheadgroup 2					Relative abundance (%)		
		Core	[M+H]^+^	AEC	Δ mmu	1	2	3
**None**		AR	653.680	C_43_H_89_O_3_	0.3	6.3	6.6	8.8
**PG**		AR	807.683	C_46_H_96_O_8_P	0.9	9.3	9.4	11.3
**DiGly**		OH-AR	993.781	C_55_H_109_O_14_	0.0	3.1	3.2	3.6
**PG**		GDGT-0	1456.325	C_89_H_180_O_11_P	1.0	0.5	1.4	0.4
		GDGT-1	1454.309	C_89_H_178_O_11_P	0.8	0.3	0.8	0.3
		GDGT-2	1452.293	C_89_H_176_O_11_P	1.5	6.6	8.8	5.8
		GDGT-3	1450.278	C_89_H_174_O_11_P	1.3	1.9	2.0	1.5
		GDGT-4	1448.262	C_89_H_172_O_11_P	1.5	5.6	4.1	4.4
		**Total**				**15**	**17**	**13**
**PG**	**Gly**	GDGT-0	1618.378	C_95_H_190_O_16_P	0.9	0.3	1.1	0.4
		GDGT-1	1616.352	C_95_H_188_O_16_P	12	2.3	3.7	2.4
		GDGT-2	1614.348	C_95_H_186_O_16_P	0.2	11.2	15.8	10.3
		GDGT-3	1612.323	C_95_H_184_O_16_P	9	6.8	7.9	6.2
		GDGT-4	1610.317	C_95_H_182_O_16_P	0.7	42.9	32.6	41.0
		**Total**				**64**	**61**	**60**
**PG**	**diGly**	GDGT-0	1780.429	C_101_H_200_O_21_P	2.2	0.1	0.2	0.2
		GDGT-1	1778.412	C_101_H_198_O_21_P	3.4	0.0	0.1	0.1
		GDGT-2	1776.399	C_101_H_196_O_21_P	0.7	1.5	1.5	1.8
		GDGT-3	1774.382	C_101_H_194_O_21_P	2.8	0.2	0.2	0.3
		GDGT-4	1772.367	C_101_H_192_O_21_P	2.2	0.6	0.3	0.7
		**Total**				**2**	**2**	**3**
**PG**	**triGly**	GDGT-0	1942.479	C_107_H_210_O_26_P	4.9	0.1	0.2	0.2
		GDGT-1	1940.468	C_107_H_208_O_26_P	0.6	0.0	0.1	0.1
		GDGT-2	1938.451	C_107_H_206_O_26_P	2.1	0.5	0.5	0.6
		GDGT-3	1936.434	C_107_H_204_O_26_P	2.9	0.1	0.1	0.2
		GDGT-4	1934.420	C_107_H_202_O_26_P	1.5	0.2	0.2	0.4
		**Total**				**1**	**1**	**1**
		**Sum AR**				**16**	**16**	**20**
		**Sum OH-AR**				**3**	**3**	**4**
		**GDGT-0**				**1**	**3**	**1**
		**GDGT-1**				**3**	**5**	**3**
		**GDGT-2**				**20**	**27**	**19**
		**GDGT-3**				**9**	**10**	**8**
		**GDGT-4**				**49**	**37**	**46**

**Table 2.** Menaquinones identified in *Oxyplasma meridianum* M1^T^ 1–3 are data of three parallel cultures of strain M1^T^. Δ mmu, measured mass – calculated mass) × 1000 as calculated for strain 1; AEC, assigned elemental composition.

	[M+H]^+^	AEC	Δ mmu	Relative abundance (%)		
				1	2	3
MK-8 : 8	717.560	C_51_H_73_O_2_	0.2	1.1	1.1	1.1
MK-7 : 7	649.498	C_46_H_65_O_2_	0.3	94.2	93.2	94.6
MK-7 : 6	651.514	C_46_H_67_O_2_	0.2	4.7	5.6	4.3

### Phylogenetic analysis

**Fig. 2.**
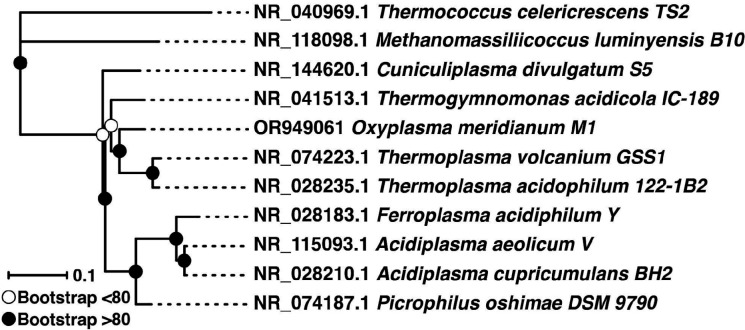
Maximum-likelihood phylogenetic tree based on 16S rRNA gene sequences of *Oxyplasma meridianum* M1^T^ and its closest phylogenetic neighbours with validly published names. Bootstrap values are based on 1000 replicates and those <80 are shown as open circles, values >80 as closed circles. Sequences were previously aligned using mafft version 7 and the resulting multiple alignment was trimmed using TrimAl 1.2rev59. The tree was reconstructed and decorated under an R programming environment using the package phangorn for the tree calculations, selecting TIM3 +I+G as best substitution model (using ModelTest pluggin within phangorn) and stochastic algorithm for tree rearrangement.

Based on its 16S rRNA gene sequence, strain M1^T^ clusters together with other archaea of the order *Thermoplasmatales*, class *Thermoplasmata*, phylum *Thermoplasmatota*. The nearest phylogenetic neighbour to strain M1^T^ is *Thermoplasma acidophilum* (90.9%), followed by *Thermogymnomonas acidicola* (90.5%) and *Thermoplasma volcanium* (90.3%) (Fig. 2). Therefore, according to the accepted boundaries for a genus (<94.5% for 16S rRNA gene sequence identity) [33], M1^T^ represents a new genus.

### Genome properties

About 239.2-fold genome coverage by Illumina reads and 23.4-fold coverage by Oxford Nanopore reads were obtained. The genome assembly resulted in one circular chromosome of 1.67 Mbp with a G+C content of 39.76 mol%. The genome annotation revealed 1725 genes with 1679 protein-coding sequences and 43 genes encoding tRNA. Analysis of the genome revealed the presence of all enzymes required for glycolysis. Genes encoding the non-phosphorylative Entner–Doudoroff pathway and the non-oxidative pentose phosphate pathway were detected. We also identified all genes encoding the TCA cycle in the genome with alpha-ketoglutarate dehydrogenase as the only exception (Table S1, available in the online Supplementary Material). Likely, the function of this enzyme is performed by 2-oxoacid:acceptor oxidoreductase. Multiple copies of genes are present in the genome, including a location in close proximity to the TCA enzyme-encoding genes. This possibility was previously considered for other organisms, including a phylogenetic neighbour of the strain M1^T^, *Picrophilus torridus* [34]. Aerobic respiration was backed up by the presence of genes encoding an NADH dehydrogenase complex, a cupredoxin-domain-containing (plastocyanin) protein, a cytochrome *cbb3*-type cytochrome C oxidase subunits I and II, and a polyferredoxin NapH superfamily (OXIME_000996 – OXIME_001001). Furthermore, cytochrome ubiquinol/*bd* terminal oxidase subunits I and II and cytochrome bc complex cytochrome b subunit encoding genes (OXIME_001706–OXIME_001707 and OXIME_000377) were identified in the genome. Moreover, we detected V-type ATP synthase subunits A, B, C, D, F, E and H in the genome of the strain M1^T^. Genes for proteolytic proteins affiliated to peptidase families M50, M13, M19, and S49, a trypsin-like peptidase, an archaeal Lon protease, a tricorn protease, and a thermopsin were found in the genome and reflect the organotrophic lifestyle of strain M1^T^. We detected genes encoding a mevalonate 3-kinase, a mevalonate 3-phosphate 5-kinase and mevalonate biphosphate decarboxylase proteins, confirming the route III of the mevalonate pathway, characteristic for *Thermoplasmatales* archaea [35,37]. Interestingly, we also identified genomic loci for hercynine oxygenase/ergothioneine biosynthesis protein EgtB (OXIME_001566) and a l-histidine *N*(alpha)-methyltransferase (OXIME_001567), both being involved into the ergothioneine pathway. Ergothioneine is a low molecular weight thiol, a derivative of histidine with a sulphur atom containing imidazole ring and was previously predicted in some archaeal genomes considering that it might be synthesized in archaea [38]. The organism encodes the CRISPR-Cas (Clusters of Regularly Interspaced Short Palindromic Repeats)-associated proteins, namely co-localized genes encoding for endoribonucleases Cas2 and Cas6, an endonuclease Cas2, type I_D protein Cas5/Csc1, Csc2, Cas4 and two copies of endonuclease Cas1 genes. To summarize, physiological, morphological and genomic features of strain M1^T^ that suggest this organism is a typical member of the order *Thermoplasmatales* (Table 3).

**Table 3.** Main characteristics of genera of the order *Thermoplasmatales* with validly published names and strain M1^T^

Characteristic	*Thermoplasma*	*Picrophilus*	*Ferroplasma*	*Acidiplasma*	*Thermogymn-omonas*	*Cuniculiplasma*	M1^T^
Cell wall/S-layer	−	+	−	−	−	−	−
Growth temperature, °C:							
Range	33–69	47–65	15–45	15/22–65	38–68	10–48	15–52.5
Optimum	67	60	35–37	45–53.5	60	37–40	40
Growth pH:							
Range	0.5–4	0–3.5	1.3–2.2	0/0.4–1.8/4	1.8–4	0.5–4	0.5–4
Optimum	1–2	0.7	1.7	1–1.6	3	1–1.2	1
Fe^2+^ oxidation	−	−	+	+	−	−	−
Anaerobic growth	+	−	±	+	−	+	−
DNA G+C content (mol%)	38–46	36	37	34–36	56	37	40

Data taken from: [7,10, 16, 31, 40,42].

**Fig. 3.**
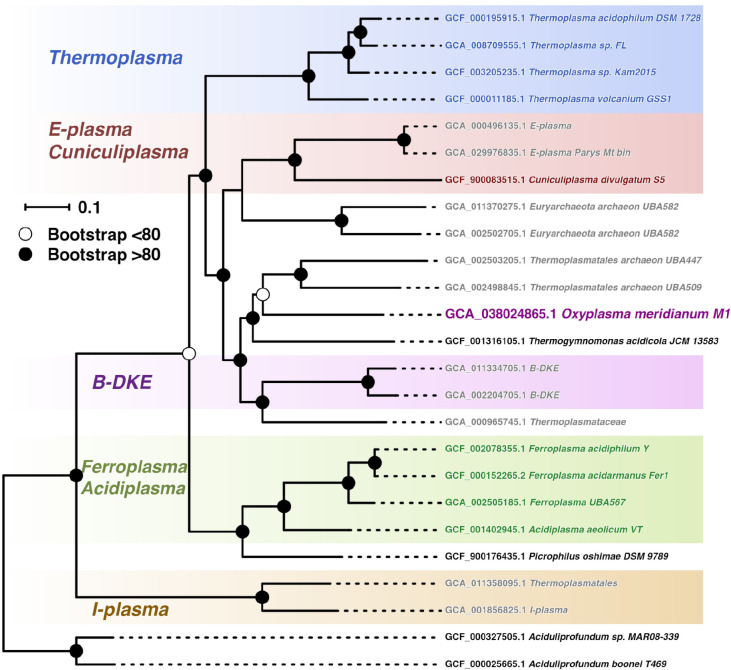
Phylogenetic tree based on 122 concatenated proteins. Tree calculation was performed using the GTDB-tk tool focusing exclusively on the order *Thermoplasmatales*, using the genus *Aciduliprofundum* as an outgroup. Bootstrap values are highlighted as closed circles (values >80), and open circles (bootstrap values <80).

The phylogenetic tree based on 122 concatenated proteins revealed that strain M1^T^ is closest to *Thermogymnomonas acidicola* among organisms with validly published names and most similar metagenome assembled genomes (Fig. 3).

dDDH (formula *d4*) showed values significantly below the threshold level of 70% and ANI calculations produced indices lower than 95–96% for strain M1^T^ and *Thermoplasmatales* archaea with validly published names (Tables S2 and S3), recommended for species delineation [39].

## Conclusion

Strain M1^T^ is a mesophilic, thermotolerant, hyperacidophilic, aerobic, organotrophic and cell-wall-lacking organism. The physiology of the organism is comparable to representatives of all genera of *Thermoplasmatales* with validly published names characterized up to date, which reflects the adaptation to specific physicochemical conditions of indigenous environments. The inability of anaerobic metabolism resembles that of *Picrophilus* and *Thermogymnomonas*, both of which were isolated from geothermal settings as well [10,31]. Electron microscopy suggested the lack of cell wall, which is also common in *Thermoplasmatales* [2,7]. Interestingly, the majority of cells had relatively small sizes (<1 µm) and possessed multiple (up to eight) filaments. The membrane lipid composition of M1^T^ was found to be rather characteristic for this group of archaea, with core lipids (GDGTs and archaeol) content being generally similar to that in *Cuniculiplasma divulgatum,* which has a similar pH and temperature range, and both having menaquinone MK 7 : 7 as the main quinone [7]. The genome of strain M1^T^ had size and G+C molar content rather typical for all known *Thermoplasmatales* and encoded proteins essential for aerobic and peptidolytic lifestyle. It should be noted that the GenBank records on 16S rRNA sequences of organisms with identities >98% to strain M1^T^ are represented by acidic environments of geothermal origin and mining regions, e.g. GenBank accession numbers, AF544219 (Iron Mountain acid mine drainage site, California, USA), KJ907756 (Michoacan, Los Azufres thermal and acidic green biofilms from a fumarole, Mexico), DQ303253 and EF441883 (floating microscopic filaments from Rio Tinto and endolithic community in the basin of Rio Tinto, Spain), and KM410353 (biofilm from subsurface sulfidic cave stream, Italy). These results imply that representatives of the genus *Oxyplasma*, similarly to phylogenetic neighbours forming the same order, are distributed across the globe in ecological niches with low pH and diverse temperatures and might be both non-thermophilic and moderately thermophilic organisms, the known property for other *Thermoplasmatales* [7]. Based on the polyphasic (genomic and phylogenomic, chemotaxonomic and physiological) analyses, strain M1^T^ is proposed to represent a novel genus and species with the name *Oxyplasma meridianum* gen. nov., sp. nov. within the family *Thermoplasmataceae*, order *Thermoplasmatales*.

## Description of *Oxyplasma* gen. nov.

*Oxyplasma* (o.xy.plas’ma. Gr. masc. adj. *oxys*, acid; Gr. neut. n. *plasma,* something shaped or moulded, N.L. neut.n.).

*Oxyplasma* a form living in acid.

Cells are lacking cell walls. Aerobic, mesophilic/thermotolerant. Organotrophic. Hyperacidophilic.

The core lipids: archaeol with trace amounts of hydroxy archaeol and glycerol dibiphytanyl glycerol tetraether lipids. The dominant quinone is MK-7 : 7.

The type species is *Oxyplasma meridianum*.

## Description of *Oxyplasma meridianum* sp. nov.

*Oxyplasma meridianum* (me.ri.di.a’num L.neut.adj. *meridianum,* southern, isolated from the South of Italy).

Cells are regular and irregular cocci about 1 µm in diameter. The temperature range for growth is 15.0–52.5 °C (optimum at 40 °C). The pH range for growth is pH 0.5–4.0 (optimum at pH 1). Grows organotrophically with tryptone, beef and yeast extracts, casamino acids, trypticase. Lipids represented mostly by archaeol with trace amounts of hydroxy archaeol and glycerol dibiphytanyl glycerol tetraether lipids (GDGT) with 0–4 cyclopentane moieties (GDGT-0–GDGT-4). The main respiratory quinone represented by menaquinone.

The type strain is M1^T^ (DSM 116605^T^=JCM 36570^T^), isolated from rock sample of Vulcano Island, Italy. The DNA G+C content of type strain is 39.76 mol%. The accession number of the strain M1^T^ 16S rRNA gene is OR949061 and for the complete genome it is CP133772.

## supplementary material

10.1099/ijsem.0.006499 Uncited Table S1.

## Abbreviations

DSMZ: German Collection of Microorganisms and Cell Cultures GmbH
TCA cycle: the citric acid cycle
TEM: transmission electron microscopy
